# Essentiality of Nfatc1 short isoform in osteoclast differentiation and its self-regulation

**DOI:** 10.1038/s41598-023-45909-3

**Published:** 2023-11-01

**Authors:** Yasuhiro Omata, Hideyuki Tachibana, Yoshimi Aizaki, Toshihide Mimura, Kojiro Sato

**Affiliations:** 1 Division of Rheumatology and Clinical Immunology, Department of Medicine, Jichi Medical University, 3311-1 Yakushiji, Shimotsuke, Tochigi 329-0498 Japan; 2 Department of Rheumatology, Akiru Municipal Medical Center, 78-1 Hikita, Akiruno, Tokyo 197-0834 Japan; 3 Department of Rheumatology and Applied Immunology, Faculty of Medicine, Saitama Medical University, 38 Moroyama, Iruma, Saitama 350-0495 Japan

**Keywords:** Osteoimmunology, Cell biology

## Abstract

During osteoclast differentiation, the expression of the transcription factor nuclear factor of activated T cell 1 (Nfatc1) increases in an autoproliferative manner. Nfatc1 isoforms are of three sizes, and only the short isoform increases during osteoclast differentiation. Genetic ablation of the whole Nfatc1 gene demonstrated that it is essential for osteoclastogenesis; however, the specific role of the Nfatc1 short form (Nfatc1/αA) remains unknown. In this study, we engineered Nfatc1 short form-specific knockout mice and found that these mice died in utero by day 13.5. We developed a novel osteoclast culture system in which hematopoietic stem cells were cultured, proliferated, and then differentiated into osteoclasts in vitro. Using this system, we show that the Nfatc1/αA isoform is essential for osteoclastogenesis and is responsible for the expression of various osteoclast markers, the Nfatc1 short form itself, and Nfatc1 regulators.

## Introduction

Osteoclasts are multinuclear giant cells responsible for bone resorption. Osteoclast impairment causes various bone diseases. For instance, osteoclast deficiency increases bone stiffness, known as osteopetrosis^1^, whereas excessive osteoclast activation leads to bone fragility that characterizes osteoporosis^2^, rheumatoid arthritis^3^, and bone metastasis^4^.

Osteoclasts can be differentiated from monocyte/macrophage lineage precursors derived from bone marrow cells^5^. Macrophage colony-stimulating factor (M-CSF), a ligand for CSF1R, regulates the proliferation, survival, and differentiation of hematopoietic cells^6^. Receptor activator of NF-κB ligand (RANKL) is a cytokine responsible for osteoclast differentiation^7–9^. RANKL-mediated stimulation of osteoclast precursors strongly upregulates the transcription factor nuclear factor of activated T cell 1 (Nfatc1) through NF-κB signaling and the c-fos pathway^10^. Nfatc1, located in the cytosol, moves into the nucleus once calcineurin phosphorylates its calcium regulatory domain, triggering the autoamplification of Nfatc1^11^. In addition, Nfatc1 induces the transcription of cathepsin K (Ctsk) ^12^ and tartrate-resistant acid phosphatase (TRAP) ^13^ in osteoclasts, both of which are important osteoclast markers. Cell–cell fusion is another unique maturation process of osteoclasts accompanied by multinucleation, which is reportedly controlled by dendrocyte-expressed seven transmembrane protein (Dcstamp) and osteoclast stimulatory transmembrane protein (Ocstamp). The lack of these genes abrogates osteoclast differentiation without affecting Nfatc1 expression^14,15^.

Whether Nfatc1 is required for osteoclast differentiation has also been investigated. Since *Nfatc1* knockout mice are embryonically lethal, likely due to the impairment of heart valve development^16,17^, it is difficult to study the function of Nfatc1 in osteoclast differentiation. However, several studies have overcome this issue. First, the essential role of Nfatc1 was demonstrated using a system in which embryonic stem cells (ES cells) differentiate into osteoclasts^10^. Transplantation of hematopoietic progenitors from *Nfatc1* whole knockout (KO) mice at E13.5 into *fos* KO mice, which lack osteoclasts, showed its essentiality in vivo^11^. The forced expression of Nfatc1 driven by the Tie2 promoter in *Nfatc1* whole KO mice rescued the formation of the heart valves during embryonic stages, leading to live births. Although the progeny appeared normal, only a few osteoclasts were formed due to the lineage-restricted expression of Nfatc1 in hematopoiesis^18^. Lastly, *Nfatc1*^*fl/fl*^ mice crossed with *Mx1-Cre* mice (*Nfatc1*^*Δ/Δ*^) revealed that Nfatc1 is required for osteoclast differentiation^19^.

While these studies support the notion that Nfatc1 is responsible for osteoclast differentiation, multiple Nfatc1 isoforms have been reported in T lymphocytes. Serfling et al. demonstrated that two distinct promoters control the transcription of *Nfatc1* mRNA variants^20,21^, and the P1 promoter initiates Nfatc1/α transcription from exon 1, whereas the P2 promoter initiates Nfatc1/β transcription from exon 2. In addition, alternative splicing and different termination signals give rise to Nfatc1/A, B, and C, which differ in the length of their C-termini. Thus, at least six Nfatc1 isoforms theoretically exist. T cell activation causes a switch from the P2 promoter to the P1 promoter, resulting in the predominant transcription of the Nfatc1 short form (Nfatc1/αA) ^21^. Similarly, the highest expression of Nfatc1/αA was observed in osteoclast differentiation compared to two longer isoforms, supposedly Nfatc1/βB and Nfatc1/βC, according to the nomenclature by Serfling’s group^21^. However, it remains unclear whether the remarkable induction of the Nfatc1/αA isoform is indispensable for osteoclast differentiation.

In this study, we confirmed that the short form of Nfatc1, but not the intermediate and long forms, is strongly induced during the differentiation of murine bone marrow-derived osteoclasts. To demonstrate the function of the short form, we generated *Nfatc1 ex1* KO mice by inserting a stop codon into exon 1 using the CRISPR/Cas9 system, abolishing the translation of the short form. Interestingly, *Nfatc1 ex1* KO mice died at E13.5. We developed a novel culture method to analyze the potential of *Nfatc1 ex1* KO hematopoietic progenitors to differentiate into osteoclasts.

## Results

### Expression of Nfatc1 short-form mRNA

**Figure 1:**
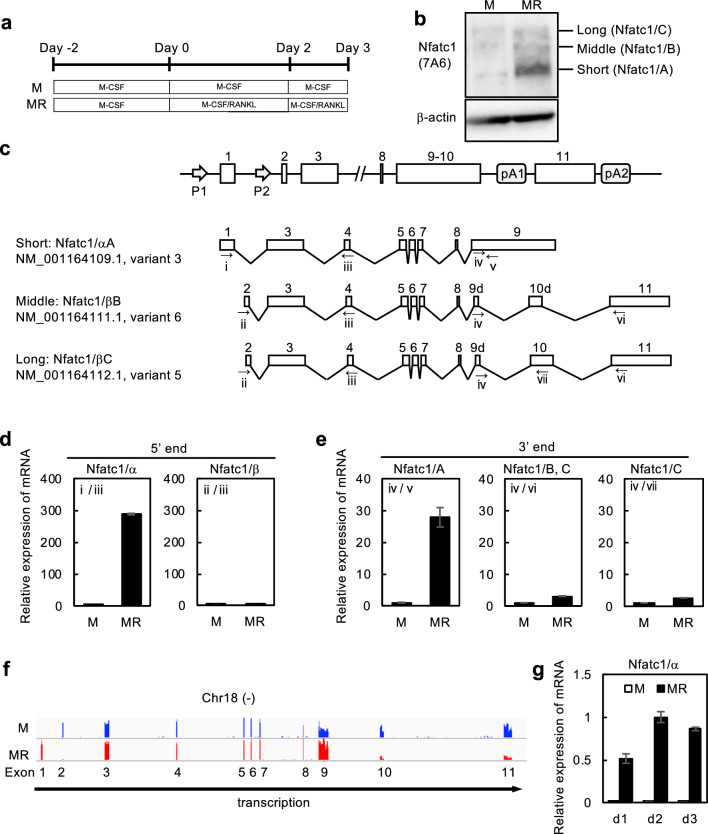
Transcription factor nuclear factor of activated T cell 1 (Nfatc1) short form expression during osteoclast differentiation. **(a)** Time course of osteoclast differentiation from bone marrow cells. Bone marrow cells were cultured in the presence of 20 ng/mL macrophage colony-stimulating factor (M-CSF) ± 100 ng/mL receptor activator of NF-κB ligand (RANKL). **(b)** Three isoforms of Nfatc1 protein on a western blot. The cells were lysed on day 3 and processed for SDS–polyacrylamide gel electrophoresis (SDS-PAGE). Signals were detected by anti-Nfatc1 antibody and anti-β-actin antibody. **(c)** Schematic illustration of Nfatc1 gene structure. Two promoters (P1 and P2) and three isoforms are shown. **(d,e)** Expression of Nfatc1 mRNA isoforms amplified by primers specific to 5′ regions (**d**) and 3′ regions (**e**). Relative expression of mRNA isoforms was analyzed by SYBR Green qRT-PCR on day 2. **(f)** Mapping data of RNA-seq reads against the C57BL6/J genome around chromosome 18 on the integrated genome browser. RNA-seq reads in culture with M-CSF alone (M) were found in exon 2, whereas RNA-seq reads in culture with M-CSF and RANKL (MR) in exon 1. **(g)** Time-dependent induction of Nfatc1/α determined by SYBR Green qRT-PCR on day 1, day 2, and day 3. Representative values are shown as mean ± SE (d, e, and g: two technical replicates). All experiments were performed at least three times using bone marrow cells from different mice. Uncropped blot images are shown in Supplementary Fig. S1.

Bone marrow cells from wild-type (WT) mice were cultured with M-CSF for 2 days, followed by culture with M-CSF and RANKL for an additional 3 days, and differentiated into TRAP-positive multinucleated cells (Fig. 1a). Nfatc1 protein isoforms, including the short, intermediate, and long forms, were detected by western blotting (Fig. 1b**,** See Supplementary Fig. S1 online); in osteoclasts, the short form was most abundantly expressed, as described by Asagiri et al^11^. These isoforms are seemingly translated from distinct mRNA isoforms consisting of different exon sets (Fig. 1c). To examine this in more detail, we performed qRT-PCR using specific primers to amplify each isoform (Fig. 1c). Expectedly, the *Nfatc1* short form mRNA (corresponding to Nfatc1/αA) was the most abundant isoform among the splicing variants (Fig. 1d, e). The intermediate and long isoforms were likely to be Nfatc1/βB and Nfatc1/βC, respectively. To confirm this, mapping RNA-seq reads onto the mouse genome revealed that reads from a culture with M-CSF were located in exon 2, whereas reads from a culture with M-CSF and RANKL were in exon 1 (Fig. 1f). Taken together, the Nfatc1 short form (Nfatc1/αA) is the major isoform induced during osteoclast differentiation. We also performed qRT-PCR to investigate the kinetics of Nfatc1 short form expression during osteoclast differentiation. The expression of the Nfatc1 short form was induced in a RANKL-dependent manner and peaked on day 2 (Fig. 1g). The substantial and specific induction of the Nfatc1 short form led us to deduce that it plays a significant role in osteoclast differentiation, which has not been proved yet.

### Generation of *Nfatc1 ex1* knockout (KO) mice

**Figure 2:**
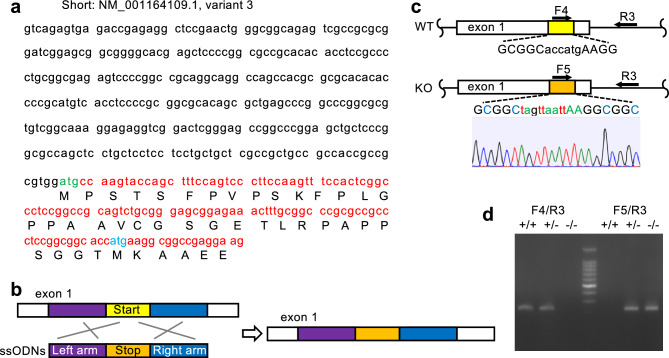
Generation of Nfatc1/αA knockout mice. **(a)** DNA sequences and amino acids of Nfatc1 short form (NM_001164109.1, variant 3) in exon 1. Sequences around the second start codon were mutated to generate *Nfatc1 ex1* knockout (KO) mice. **(b)** A strategy of genome editing. Single-stranded oligo DNA (ssODNs) with a stop codon (orange) between two arms (purple and blue) were synthesized and supplied to repair a double-strand break. **(c)** Genomic illustration of *Nfatc1 ex1* KO mice. An insert containing a stop codon in exon 1 abolishes translation of the Nfatc1 short form. **(d)** Genotyping of genomic DNA prepared from tail tips using primer F4 for wild-type (WT) allele or F5 for KO allele. The primer pair (F4 and R3) amplified 226 bp fragments of the WT genome, whereas the primer pair (F5 and R3) did 225 bp fragments of the KO genome. An uncropped image of the gel is shown in Supplementary Fig. S2.

We generated *Nfatc1 ex1* KO mice to test the above hypothesis using the CRISPR/Cas9 system. Inactivation of the first start codon (ATG) of the NCBI reference sequence (NM_001164109.1, variant 3, Nfatc1/αA) raised the concern that the second ATG might function as a translation initiation site of the Nfatc1 short form (Fig. 2a). In order to target the second start codon, we designed single-stranded oligo DNA (ssODN) including a stop codon between two arms (Fig. 2b), and injected it with guide RNA and Cas9. As a result, the second ATG codon in exon 1 was replaced with a 9 bp sequence containing a stop codon (Fig. 2c). Founder mice crossed with WT mice delivered the same number of WT and heterozygous siblings, according to the Mendelian ratio. These mice did not have any significant defects (data not shown). Genotyping was performed using PCR primers complementary to WT or KO alleles (Fig. 2d**,** See Supplementary Fig. S2 online). Mating between *Nfatc1 ex1*^+*/-*^ mice gave rise to WT and heterozygous siblings at a ratio of 1:2 (Table 1). We did not observe any developmental failure at E11.5 but found that *Nfatc1 ex1*^*−/−*^ mice were embryonically lethal by E13.5, slightly earlier than *Nfatc1* whole KO mice^16,17^.

**Table 1:** Number of embryos generated from crossing between heterozygous mice.

	+ / +	+ /−	−/−	Total
E10.5	10	13	8	31
E11.5	12	35	16	63
E12.5	4	8	5 (1)	17
E13.5	24	43 (2)	8 (7)	75
Total	50	99 (2)	37 (8)	186

The numbers of dead embryos are shown in parentheses.

### Osteoclast differentiation from hematopoietic progenitors

**Figure 3:**
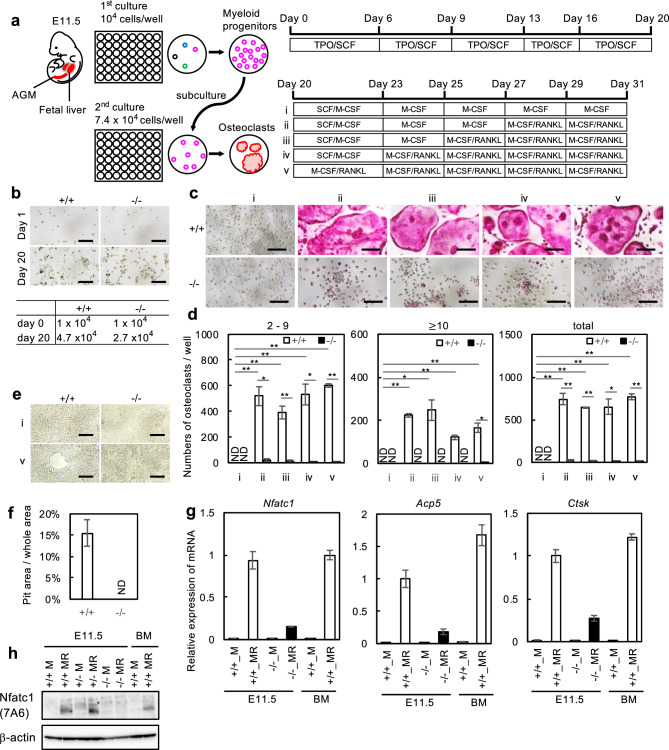
Loss of osteoclasts in a culture of hematopoietic progenitors from *Nfatc1 ex1* KO mice. **(a)** First, hematopoietic progenitors collected from the fetal liver and aorta-gonad-mesonephros (AGM) at E11.5 were cultured with 100 ng/mL thrombopoietin (TPO) and 10 ng/mL stem cell factor (SCF) on fibronectin-coated 48 well plates for 20 days. Then, the proliferating cells were harvested, reseeded, and cultured with 10 ng/mL SCF, 20 ng/mL M-CSF, and 100 ng/mL RANKL for 11 days for osteoclast differentiation. **(b)** Representative images of cultured hematopoietic progenitors from WT and KO mice (upper). Number of hematopoietic progenitors on day 0 and day 20 (bottom). Scale bars, 100 μm. **(c)** Tartrate-resistant acid phosphatase (TRAP) -positive cells in culture of hematopoietic progenitors. Hematopoietic progenitors from WT and KO fetuses at E11.5 were cultured according to the condition shown in (**a**). The cells were stained on day 31. Scale bars, 100 μm. **(d)** The number of osteoclasts formed in different culture periods with RANKL for WT and KO hematopoietic progenitors. TRAP-positive cells were classified with a nuclear number and counted. Four experiments were performed independently using hematopoietic progenitor cells from different embryos. Representative data are shown as mean ± SE (n = 3 technical replicates). Statistical significance between the number of KO and WT osteoclsts was determined using Student’s *t*-test (** p < 0.01, * p < 0.05), whereas that in culture conditions was determined using the Tukey Kramer test (** p < 0.01). **(e)** Bone resorption assay. Hematopoietic progenitor cells were cultured on phosphate calcium-coated 48 well plates for 26 days under the condition of (i) or (v). Representative images of the pits are shown. Scale bars are 100 μm. **(f)** Pit areas were measured at 5 different areas and shown as mean ± SE. **(g)** Relative mRNA expression of *Nfatc1/α*, *Acp5*, and *Ctsk* in culture condition (iii) hematopoietic progenitors on day 27. All experiments were performed three times using total RNA from hematopoietic progenitors of different embryos. Representative values are shown as mean ± SE (n = 2 technical replicates). **(h)** Western blots of Nfatc1 and β-actin. Cultured hematopoietic progenitors on day 27 of condition (iii) and bone marrow cells on day 3 were lysed and subjected to SDS-PAGE. Uncropped blot images are shown in Supplementary Fig. S1.

To analyze the ability of *Nfatc1 ex1*^*−/−*^ to differentiate into osteoclasts, we cultured dissociated fetal liver cells from E13.5 *Nfatc1 ex1*^*−/−*^ fetuses, in which hematopoietic progenitors were supposed to undergo rapid proliferation. Although WT fetal liver cells differentiated into large osteoclasts, *Nfatc1 ex1*^*−/−*^ fetal liver cells did not survive when cultured under the same conditions (data not shown). Therefore, we next utilized hematopoietic progenitors derived from the aorta-gonad-mesonephros (AGM) regions and the livers at E11.5. These cells were maintained on fibronectin-coated dishes in HemEx Type-9A supplemented with stem cell factor (SCF) and thrombopoietin (TPO) (Fig. 3a), which were exclusively developed for the culture of hematopoietic stem cells^22^. This method enabled us to obtain sufficient hematopoietic progenitors for studying their potential to differentiate into osteoclasts. Both hematopoietic progenitors from WT and *Nfatc1 ex1*^*−/−*^ mice at E11.5 did not start growing until day 7 but proliferated rapidly thereafter. On day 20, these proliferating hematopoietic progenitors were harvested. We found that the number of hematopoietic progenitors from WT and *Nfatc1 ex1*^*−/−*^ mice increased approximately fivefold and threefold, respectively (Fig. 3b). Second, the suspension from the first culture was reseeded and cultured for an additional 11 days. Usually, 3–4 days of exposure to RANKL is sufficient for mouse osteoclast differentiation from bone-marrow cell-derived monocyte/macrophage lineage cells. However, as the optimal condition in which osteoclasts differentiate from hematopoietic progenitor cells was unknown, we tested various periods of exposure to RANKL (Fig. 3a). In essence, multinucleated cells appeared 2–3 days after RANKL stimulation under all the conditions tested. While an increase of osteoclasts and enlargement were observed microscopically along with the time course, there were no significant differences in the total number of osteoclasts differentiated from WT cells under the conditions tested on day 31 (Fig. 3d). However, as prolonged treatment with RANKL tended to cause the detachment of larger osteoclasts from the plates before TRAP staining, there seemed to remain fewer TRAP-positive cells (nuclear number > 10) in culture conditions (iv) and (v) than in conditions (ii) and (iii) (Fig. 3d). Contrastingly, *Nfatc1 ex1* KO cells barely formed multinuclear osteoclasts under any of the conditions (Fig. 3c, d). In addition, whereas hematopoietic progenitors from WT mice cultured under condition (v) showed bone matrix resorption activity, those from *Nfatc1 ex1* KO did not (Fig. 3e, f).

We also examined the relationship between seeding density and osteoclast differentiation. A dilution series of embryonic hematopoietic progenitors prepared from the first culture was cultured with SCF, M-CSF, and RANKL under culture conditions (ii). The number of TRAP-positive osteoclasts differentiated from WT embryonic hematopoietic progenitors decreased in descending order, but *Nfatc1 ex1* KO embryonic hematopoietic progenitors developed a very small number of fused TRAP-positive osteoclasts, regardless of seeding density (Supplementary Fig. S3 online).

Next, the expression of the short isoform of Nfatc1 in osteoclasts differentiated from embryonic hematopoietic progenitors under condition (iii) on day 27 was examined by qRT-PCR. The short form mRNA expression during the culture of WT hematopoietic progenitors with M-CSF and RANKL was much higher than in the culture with M-CSF (Fig. 3g). In addition, the induction of Nfatc1/βB and Nfatc1/βC in this culture system by RANKL was substantially less than that of Nfatc1/αA (Supplementary Fig. S4 online), as in the case of bone-marrow derived osteoclasts (Fig. 1d, e). The expression of the *Nfatc1* short isoform in the culture of *Nfatc1 ex1*^*−/−*^ hematopoietic progenitors with M-CSF and RANKL was lower than that in the culture of WT hematopoietic progenitors (Fig. 3g, left panel). At the protein level, the short form could be distinctly detected in the WT culture but not in *Nfatc1 ex1*^*−/−*^ cultures (Fig. 3h). The expression levels of osteoclast marker genes were tested under the same culture conditions. Expression of *Acp5* and *Ctsk* was found in osteoclasts differentiated from WT embryonic hematopoietic progenitors cultured with M-CSF and RANKL, but the expression of these genes did not increase adequately in the culture of hematopoietic progenitors from *Nfatc1 ex1* KO mice (Fig. 3g, middle and right panels).

### Expression profiles of genes in osteoclasts

**Figure 4:**
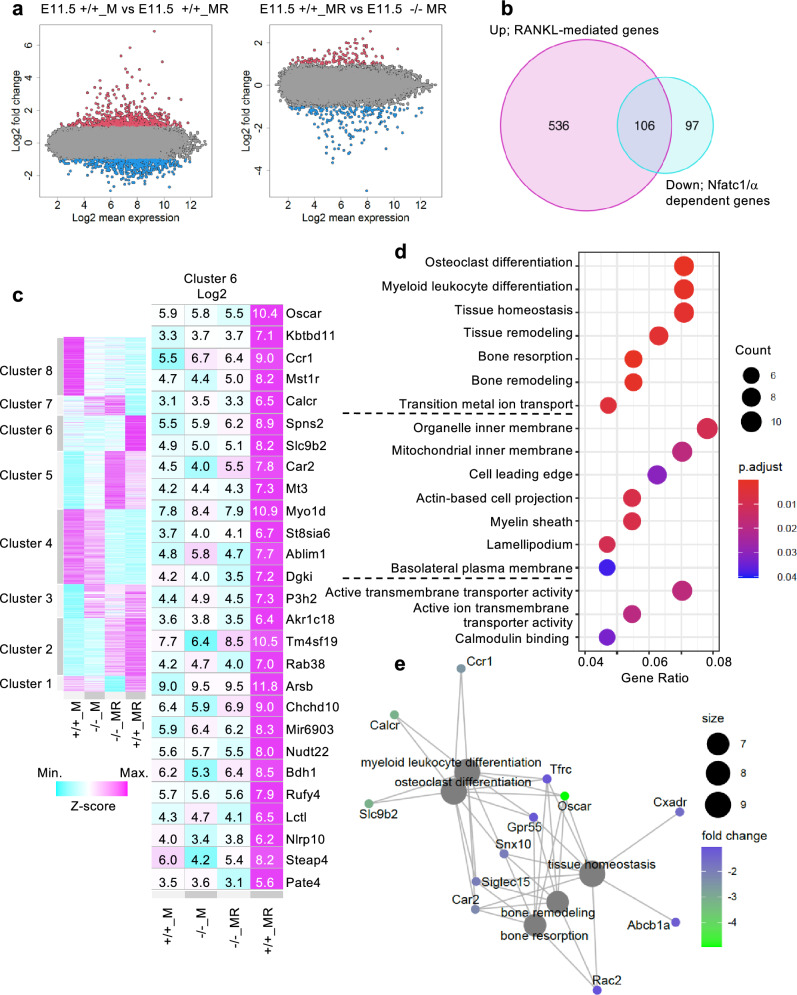
Altered gene expression in hematopoietic progenitors from *Nfatc1 ex1* KO mice cultured in the presence of M-CSF ± RANKL. Hematopoietic progenitors at E11.5 from WT and homozygous were cultured in condition (iii). The total RNA extracted on day 27 was analyzed using Gene Chip. **(a)** MA plots of log_2_ fold-change from 20,763 genes. Log_2_ fold-change > 1.0 (red), log_2_ fold-change < −1.0 (blue). Left: hematopoietic progenitors from WT cells cultured with M-CSF vs. M-CSF and RANKL. Right: culture with M-CSF and RANKL of hematopoietic progenitors from WT embryos vs. homozygous embryos. **(b)** Venn diagram shows the numbers of genes increased by culturing WT hematopoietic progenitors with RANKL and decreased by the loss of Nfatc1/α. **(c)** A heatmap of 1340 genes identified as RANKL-mediated genes and/or Nfatc1α-dependent genes (log_2_ fold change > 1.0 and/or < −1.0) were classified into eight clusters. An extension of Cluster 6 listed 27 genes (log_2_ fold-change > 2.0 and/or < −2.0). **(d)** Dot plots of significantly enriched GO terms from 136 genes in Cluster 6. Gene ratios and counts of more than six were displayed in each ontology category. **(e)** Gene-concept network of enriched GO terms in Cluster 6. The GO terms are spotted in a gray-filled circle corresponding to their size. Genes are colored based on log_2_ fold change (E11.5 + / + _MR vs. E11.5 −/−_MR).

To investigate the effect of RANKL and the Nfatc1 short form on gene expression profiles, we performed a gene chip analysis using total RNA isolated from cultured cells under condition (iii) on day 27. Alterations in gene expression mediated by RANKL and the lack of the Nfatc1 short form are shown as an MA plot separately (Fig. 4a). The number of genes exhibiting a RANKL-mediated increase and a decrease caused by a lack of the Nfatc1 short form was 106 (Fig. 4b). A heatmap displaying 1340 genes with altered expression levels (log_2_ fold change > 1.0 or < −1.0) caused by RANKL treatment and/or lack of Nfatc1/αA, which were classified into eight clusters, is shown in Fig. 4c (left). RANKL-mediated and Nfatc1/αA-dependent 136 genes were found in Cluster 6. Remarkably altered 27 genes (log_2_ fold change > 2.0, log_2_ fold change < −2.0) are listed in a comprehensive heatmap (Fig. 4c, right). Interestingly, the addition of RANKL to WT hematopoietic progenitors increased the expression levels of *actin-binding LIM protein 1 (Ablim1)*, *osteoclast-associated receptor (Oscar)*, and *kelch repeat and BTB (POZ) domain containing 11 (Kbtbd11)*, which are required for osteoclast differentiation from bone marrow cells^23–25^, and the lack of the Nfatc1 short form decreased the expression of these genes. In contrast, expression levels of *Dcstamp* and *Ocstamp*, which are responsible for the fusion of migrating monocytes and macrophages^14,15^, did not substantially decrease (Table 2, upper rows), similar to the marker genes for monocytes and osteoclast precursors (Table 2, lower rows).

**Table 2:** Expression of marker genes.

Cell type and function	Genes	E11.5 +/+	E11.5 −/–	E11.5 +/+	E11.5 −/−
		M	M	MR	MR
Osteoclast fusion	Dcstamp	4.51	4.39	10.37	9.80
	Ocstamp	4.83	4.21	9.38	8.84
Monocytes and macrophages	Cd80	8.34	8.96	8.28	8.69
	Csf1r	12.20	12.04	11.80	11.44
	Itgam	11.09	10.92	10.73	10.98
	Lyz2	12.72	12.56	12.31	12.31
	Tnfrsf11a	8.68	8.51	9.50	9.41

To estimate the function of alterations in gene expression, gene ontology (GO) analysis was performed for 136 genes in Cluster 6 (see Supplementary Table S1 online). Significantly enriched GO terms for biological process (BP), cellular components (CC), and molecular function (MF) were plotted along with gene counts and their ratios in each category (Fig. 4d). In addition, gene-concept network analysis of 136 genes from Cluster 6 predicted a network for osteoclastogenesis associated with “osteoclast differentiation,” “myeloid leukocyte differentiation,” “tissue homeostasis,” “bone remodeling,” and “bone resorption” (Fig. 4e).

**Figure 5:**
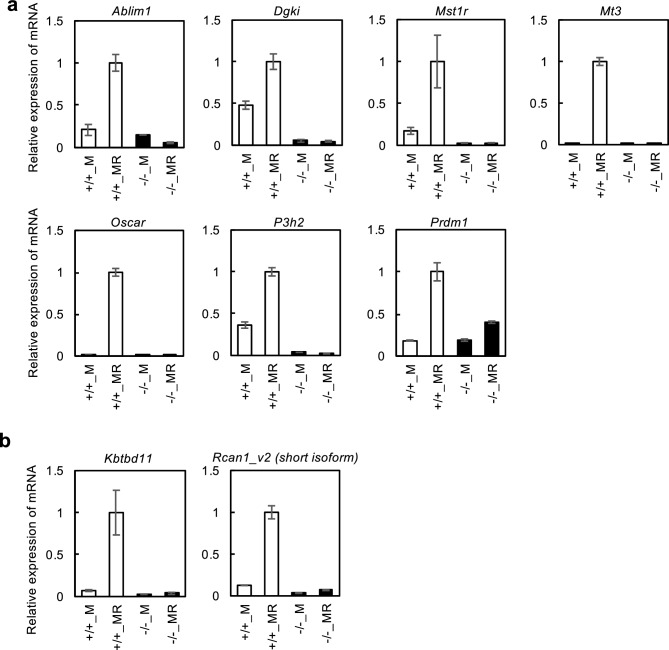
Decrease of gene expression in the hematopoietic progenitors from *Nfatc1 ex1* KO mice. SYBR Green qRT-PCR was performed using total RNA isolated from cultured hematopoietic progenitors on day 27 of culture condition (iii). **(a)** Relative mRNA expression of *Ablim1*, *Dgki*, *Kbtbd11*, *Mst1r*, *Mt3*, *Oscar*, *P3h2*, and *Prdm1* that are induced in osteoclast differentiation. **(b)** Relative mRNA expression of *Kbtbd* and *Rcan1 v2* (short isoform) that inhibit osteoclast differentiation. All experiments were performed twice using total RNA from hematopoietic progenitors of different embryos. Representative values are shown as mean ± SE (n = 2 technical replicates).

Finally, qRT-PCR was performed against *Ablim1, diacylglycerol kinase iota (Dgki)*, *macrophage-stimulating 1 receptor (Mst1r)*, *metallothionein 3 (Mt3)*, *Oscar*, *prolyl 3-hydroxylase 2 (P3h2)*, and *PR domain containing 1 with ZNF domain (Prdm1)* to confirm the alteration of gene expression in the Gene Chip analysis. All these genes showed an increase in culture with M-CSF and RANKL from WT mice and a decrease in culture from *Nfatc1 ex1*^*−/−*^ mice (Fig. 5a). The protein level expression of Ablim1, Oscar, and Prdm1 was confirmed in osteoclasts developed from bone marrow cells (Supplementary Fig.S5 online). In addition, *kelch repeat and BTB (POZ) domain containing (Kbtbd11)* and *regulator of calcineurin 1 (Rcan1)* showed similar alterations (Fig. 5b).

## Discussion

Conventional *Nfatc1* whole KO mice showed anemia at E12.5, abnormal heart valve development, and died by E14.5^16,17^. Our *Nfatc1 ex1* KO mice died by E13.5, strongly indicating that the functions of the Nfatc1 short form could not be compensated by those of the intermediate and long isoforms.

It was surprising that *Nfatc1 ex1* KO mice did not have a longer life span than KO mice. In this regard, the function of Nfatc1 has recently been reported to be governed by transcriptional regulation driven by different promoters and a post-translational modification, sumoylation^26,27^. Sumoylated human NFATc1/C translocates to the nuclear bodies and causes histone deacetylation^26^, negatively regulating the transactivation of NFATc1/C. Nfatc1/B and Nfatc1/C have two more consensus amino acid sequences for sumoylation than Nfatc1/αA. Thus, Nfatc1/B and Ntatc1/C may have opposite functions to Nfatc1/αA with respect to the transcription of some genes. This may explain the shorter life span of *Nfatc1 ex1* KO mice.

Dissociated cells from fetal livers at E13.5 did not survive with M-CSF, suggesting that the Nfatc1 short form is required for the sufficient differentiation of monocyte/macrophage precursors in the liver. Thus, we developed a novel in vitro osteoclast differentiation system to analyze the effect of the *Nfatc1* short form KO. During definitive hematopoiesis, hematopoietic progenitors from AGM migrate into the fetal liver after E11.5 and rapidly initiate proliferation^28–30^. They also have the potential to differentiate into macrophages^31^. In this study, we applied a serum-free culture medium with SCF and TPO that was developed for the culture of hematopoietic stem cells^22^ to culture hematopoietic progenitors derived from the AGM region and liver of *Nfatc1 ex1* KO mice and obtained a sufficient number of viable hematopoietic progenitors (Fig. 3a, b). Regardless of the incubation duration with SCF and M-CSF, WT hematopoietic progenitors differentiated into osteoclasts (Fig. 3c, d). We also succeeded in amplifying *Nfatc1 ex1* KO hematopoietic progenitors using this method, although the number of cells obtained was smaller than that of WT cells. Thus, we can safely say that the KO mice maintained definitive hematopoiesis, and hematopoietic progenitors could differentiate into monocyte/macrophage lineage cells, judging from the similar levels of monocyte markers such as *Cd80 antigen*^32^, *Integrin alpha M (Itgam)*^10^, *Colony-stimulating factor 1 receptor (Csf1r)*^33^, *Lysozyme 2 (Lyz2)*^34^, and a marker of osteoclast precursor cells, *Tnfrsf11a*^35^ in WT and *Nfatc1 ex1* KO cells (Table 2).

Regarding hematopoiesis, Barahona de Brito et al. recently reported that *Vav-Cre Nfatc1*^*fl/fl*^ mice had fewer hematopoietic stem cells and progenitor cells (HSPC) in the bone marrow^36^. Interestingly, *Vav-Cre Nfatc1 P2*^*fl/fl*^ mice did not exhibit impaired hematopoiesis. They claimed that transcripts induced by the P1 promoter could compensate for Nfatc1 isoforms transcribed by the P2 promoter. However, our data suggest that the Nfatc1 short form induced by the P1 promoter is more important for the differentiation of hematopoietic progenitors than P2 promoter-driven transcripts.

Our new culture method allowed us to evaluate the osteoclastogenesis potential of *Nfatc1 ex1* KO cells and compare their gene expression profiles with those of WT osteoclasts. *Nfatc1 ex1* KO cells did not become multinucleated after RANKL stimulation despite substantial induction of Ocstamp and Dcstamp, which are known to be crucial for multinucleation (Table 2). Reduction of *Ablim1* (Fig. 4c) might render osteoclast precursor cells unable to fuse as *Ablim1* is required for cell migration^23^. Expression of *Myo1d* encoding myosin 1d was also lower in KO cells than in WT cells. Although the role of *Myo1d* in osteoclast differentiation has not yet been elucidated, as myosin functions as a motor protein with actin filaments^37^, it may be involved in migration, fusion, and the formation of the actin-rich sealing zone in terminally differentiated osteoclasts. Of note, cathepsin, TRAP, and protons are secreted into the resorption lacuna through the ruffled border surrounded by the sealing zone for bone degradation^38^.

Our results indicate that various osteoclast marker genes, such as *Acp5* and *Ctsk*, are also Nfatc1/αA-dependent (Fig. 3g). Acp5 is a histological marker of osteoclasts with osteoclastic activity^39^, whereas Ctsk degrades matrix collagen and proteolyzes Acp5^40^. In addition, Oscar is a cell surface molecule that increases significantly during osteoclast differentiation^24^. We previously showed by promoter analysis that Nfatc1 directly controls *Oscar* expression^41^. As we barely detected *Oscar* expression in *Nfatc1 ex1* KO cells (Fig. 5a), we can safely say that the transcription of *Oscar* is exclusively dependent on Nfatc1/αA.

Other functional molecules are also regulated by Nfatc1/αA. Mst1r is a receptor for macrophage-stimulating proteins (MSP) that activates Src, which then causes cytoplasmic contraction and ruffled border formation, ultimately facilitating bone resorption^42,43^. Nishikawa et al. reported that the transcriptional repressor Prdm1 suppresses the expression of anti-osteoclastogenic genes, such as *Interferon regulatory factor 8 (Irf8)* and *v-Maf musculoaponeurotic fibrosarcoma oncogene family protein B (MafB)*^44^. We found that the expression levels of *Mst1r* and *Prdm1* were substantially lower in *Nfatc1 ex1* KO mice than in WT mice (Fig. 5a).

Thus, we demonstrated that Nfatc1/αA positively regulates the transcription of various genes important for osteoclast differentiation and function, among which Nfatc1/αA itself is involved (Fig. 3g). If uncontrolled, such a positive feedback mechanism can lead to a catastrophic cellular outcome. Thus, Nfatc1/αA is likely to regulate the negative feedback loop(s) as well. Among such candidates is Kbtbd11, which controls osteoclastogenesis through Nfatc1 ubiquitination^25^. Another candidate is Rcan1, which inhibits the nuclear translocation of Nfatc1 by blocking calcineurin during vascular endothelial cell differentiation^45^. This molecule has also been implicated in the regulation of osteoclast differentiation^46^. The expression levels of both molecules were also highly downregulated in *Nfatc1 ex1* KO cells. Interestingly, Rcan1 has both long and short isoforms. The expression of the long form is constitutive, whereas that of the short form is inducible^47^, similar to that of Nfatc1^20,21^. We confirmed that the expressions of both *Kbtbd11* and the *Rcan1* short form at the mRNA level were induced solely in WT osteoclasts (Fig. 5b).

**Figure 6:**
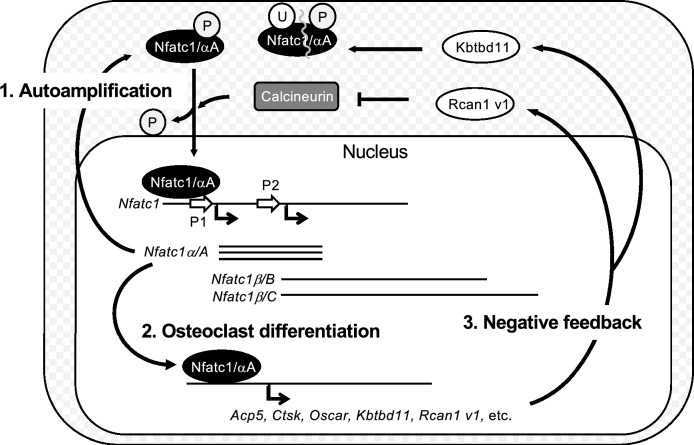
Osteoclast differentiation promoted by Nfatc1/αA and inhibited by a feedback loop. Three roles for Nfatc1 in osteoclast differentiation. (1) Calcineurin activated by Ca^2+^ signaling dephosphorylates Nfatc1 in the cytosol, which translocates into the nucleus. Nfatc1/αA amplifies its expression by binding to promoter P1, constituting a positive feedback loop. (2) Nfatc1/αA induces the expression of other genes that are important for osteoclast differentiation. (3) Nfatc1/αA also induces the expression of its negative regulators. The short form of Rcan1 inhibits calcineurin, whereas Kbtbd11 is involved in ubiquitin-dependent Nfatc1 proteolysis. Thus, Nfatc1/αA regulates both accelerators and brakes for fine-tuning osteoclast differentiation.

The results of our transcriptome analysis were similar to those of Aliprantis et al., in which CKO mice (*Mx-Cre; Nfatc1*) were used^19^. Thus, the phenotype of *Nfatc1 ex1* specific KO was similar to that of *Nfatc1* whole KO cells in osteoclast differentiation, indicating that Nfatc1/αA alone is capable of mediating osteoclastogenesis, which cannot be compensated for by the longer isoforms. The multiple roles of Nfatc1/αA in osteoclast differentiation are summarized in Fig. 6.

This study has a few limitations. Although we suspect that the proliferation of *Nfatc1 ex1* KO hematopoietic stem cells is defective, the stage(s) at which it is impaired remains unclear. The migration of hematopoietic progenitors from AGM into the fetal liver may also be impaired. In addition, we have clarified which molecules are induced by the Nfatc1 short form among various osteoclast markers, but it is unclear whether they are direct targets. Chromatin immunoprecipitation assay would be informative if Nfatc1 short form specific antibodies were available. In order to perform more detailed analysis of molecular mechanisms by which Nfatc1/αA regulates osteoclast differentiation, the establishment of the inducible gene knockout system by making *Nfatc1 ex1*^*fl/fl*^ mice might be necessary. In that case, we must be careful in choosing suitable Cre mice to mate with them. For example, *Mx-Cre* mice may not be suited for detailed analyses because Type I interferon signaling has been shown to strongly inhibit osteoclast differentiation ^48^. Establishing suitable in vitro and in vivo models for comprehensively analyzing the roles of Nfatc1/αA in osteoclastogenesis is an important task for the future.

In summary, we generated *Nfatc1 ex1* KO mice and investigated the function of the Nfatc1 short form during osteoclast differentiation. Although *Nfatc1 ex1* KO mice were embryonically lethal, we successfully evaluated the ability of the cells derived from the mice to differentiate into osteoclasts using newly developed culture methods. *Nfatc1 ex1* KO cells did not differentiate into osteoclasts, and many essential molecules involved in osteoclastogenesis were not induced in these cells, indicating that Nfac1/αA is critical for osteoclast differentiation.

## Materials and methods

### Mice

*Nfatc1 ex1* KO mice with a stop codon inserted in exon 1 were generated by genome editing. CRISPR RNA (crRNA; GCCCTCCGGCGGCACCATGAAGG) was synthesized to target the second start codon in Nfatc1 exon 1 using the CRISPR/Cas9 target online predictor (CCTop, http://crispr.cos.uni-heidelberg.de)^49^. crRNA was annealed with trans-activated crRNA (tracrRNA) to form guide RNA (gRNA). Digestion efficiency was tested by incubating the gRNA and Cas9 with fragments of the target DNA prepared by PCR amplification. Cleaved DNA fragments were detected on an agarose gel. Next, 129 bp ssODN with a stop codon between the two homology arms was synthesized (Fig. 2b). The gRNA, Cas9, and ssODNs were injected into fertilized eggs derived from C57BL/6J^50^. The Cas9 target region from nine F0 mice was amplified using PCR and sequenced. An insertion including a stop codon was detected in two F0 mice, which were crossed with C57BL/6J mice purchased from Charles River Laboratories Japan (Yokohama City, Kanagawa, Japan). Pregnant mice were euthanized by CO_2_ exposure. Embryos were decapitated, and then hematopoietic progenitor cells were collected from the fetal liver and AGM. All mice were maintained under specific pathogen-free conditions. Animal experiments were approved by the Animal Study Committee of Jichi Medical University (No. 19018-01), and all methods were performed in accordance with the relevant guidelines and regulations. All animal studies were performed according to ARRIVE guidelines (https://arriveguidelines.org/arrive-guidelines/results).

### Genotyping

Tail tips from mice were digested with proteinase K (Takara Bio Inc. Kusatsu, Shiga, Japan) at 56 °C for 3 h. Dilution of the crude solution was used as a PCR template, amplified with KOD FX DNA polymerase (TOYOBO, Osaka, Japan), and primers specifically designed to detect WT and KO alleles. The primer sequences are shown below. F4: 5′-TCC GGC GGC ACC ATG AAG GC-3′, F5: 5′-GCG GCT AGT TAA TTA AGG CGG C-3′, R3: 5′-CCG GGG AAA CAC CAG GGG GA-3′. The PCR conditions were set as follows: 94 °C for 2 min, followed by 35 cycles of 98 °C for 10 s, 63 °C for 30 s, and 68 °C for 30 s.

### Bone marrow cell culture

Osteoclast differentiation from bone marrow cells has been described previously^51^. Briefly, bone marrow cells (2 × 10^5^ cells/well) were cultured in α-minimum essential medium (Thermo Fisher Scientific, Waltham, MA, USA) containing 10% fetal bovine serum (Sigma-Aldrich Inc. St. Louis, MO, USA), 2.2 mg/mL sodium hydrogen carbonate (Fujifilm Wako Pure Chemical Corporation, Osaka, Japan), and penicillin–streptomycin (50×; FUJIFILM Wako Pure Chemical Corporation) in 24 well plates. Bone marrow cells were cultured with murine M-CSF (20 ng/mL; PeproTech, Cranbury, NJ, USA) for 2 days. For the next 3 days, murine M-CSF (20 ng/mL) and murine RANKL (100 ng/mL; PeproTech) were added.

### Culture of embryonic hematopoietic progenitors

The fetal liver and AGM were dissected microscopically from embryos at E11.5 using forceps and then dissociated into cells by applying friction between the frosted ends of slide glasses. The suspension in HemEx Type-9A (Cell Science & Technology Institute Inc. Sendai, Miyagi, Japan) was seeded at 10^4^ cells/well in 48 well plates coated with human fibronectin (40 μg/mL, Corning Inc, Corning, NY, USA) and cultured in serum-free conditions in the presence of mouse TPO (100 ng/mL; PeproTech) and mouse SCF (10 ng/mL; PeproTech) for 20 days, during which half of the medium was changed every 3 days after 7 days of culture. Proliferating hematopoietic progenitors were harvested using a pipette on day 20 and suspended in α-minimum essential medium. Suspensions seeded at a concentration of 7.4 × 10^4^ cells/well in 48 well plates were cultured with murine SCF (10 ng/mL), murine M-CSF (20 ng/mL), and murine RANKL (100 ng/mL) for osteoclast differentiation.

### TRAP staining

Cultured cells were fixed with 10% formalin neutral buffer solution (FUJIFILM Wako Pure Chemical Corporation) at 27 °C for 10 min. Color development was performed using a TRAP staining kit (Cosmo Bio, Tokyo, Japan). Stained cells were immersed in PBS and observed using an IX50 inverted microscope (Olympus, Tokyo, Japan) with a 10× objective and bright field (BF) filter at 27 °C. Images were acquired with an Advancam-HD-2 s camera (Advan Vision, Tokyo, Japan).

### Bone resorption assay

Bone marrow cells and hematopoietic progenitors were cultured on calcium phosphate-coated plates (PG Research, Tokyo, Japan). Hematopoietic progenitors were cultured with murine M-CSF (20 ng/mL) and murine RANKL (100 ng/mL) for 26 days. After removing the cells with 5% hypochlorous acid, five fields per condition were captured. Pit areas were measured using ImageJ 1.53K (National Institute of Health, Bethesda, MD, USA) ^52^.

### qRT-PCR

Total RNA from cultured bone marrow cells or embryonic progenitors was extracted using the RNeasy Micro Kit (Qiagen, Venlo, Netherlands). The concentration of total RNA was determined using a NanoDrop One (Thermo Fisher Scientific). The PrimeScript™ II 1st strand cDNA Synthesis Kit (Takara) was used for the reverse transcription of total RNA (300 ng). Diluted cDNA was amplified using Power SYBR™ Green PCR Master Mix (Thermo Fisher Scientific) or TaqMan™ Fast Advanced Master Mix (Applied Biosystems) in StepOnePlus Real-Time PCR (Applied Biosystems). The relative mRNA expression levels were quantified using the ΔΔCt method. Primer sequences designed for SYBR Green detection are listed in Supplementary Table S2 online. TaqMan Gene Expression Assays were used to detect the mRNA levels of *Acp5* (Mm00475698_m1; Thermo Fisher Scientific), *Ctsk* (Mm00484039_m1; Thermo Fisher Scientific), and *Gapdh* (Mm99999915_g1; Thermo Fisher Scientific).

### Western blots

Cultured murine cells were lysed in RIPA buffer (Nacalai Tesque, Kyoto, Japan). Protein concentration was determined using a bicinchoninic acid (BCA) assay (Thermo Fisher Scientific). The denatured lysate mixed with Novex™ Tris–Glycine SDS Sample Buffer (2×; Thermo Fisher Scientific) was loaded onto Novex™ WedgeWell™ 4–12% Tris–Glycine gels (Thermo Fisher Scientific) for electrophoresis. The gels were electroblotted onto a PVDF membrane using iBlot 2 PVDF mini stacks (Thermo Fisher Scientific). Membranes were blocked with Blocking One (Nacalai Tesque) for Nfatc1 detection and with 5% skim milk (FUJIFILM Wako Pure Chemical Corporation) for β-actin. The membranes were incubated with primary antibodies overnight at 4 °C, followed by incubation with secondary antibodies at room temperature for 1 h. ECL Prime Western Blotting Detection Reagent (GE Healthcare Bioscience, Chicago, IL, USA) was added to the membranes, and the chemiluminescent signal was detected using a CCD camera (Vilber, Collégien, France). The primary antibodies used were mouse monoclonal antibodies against Nfatc1 (Santa Cruz Biotechnology, Dallas, TX, USA, sc-7294, 1:1000 dilution) and β-actin (Sigma-Aldrich, A1978, 1:2000 dilution). The secondary antibody was an ECL peroxidase-labeled anti-mouse antibody (GE Healthcare Bioscience. 1:10,000 dilution).

### RNA-seq

The quality of each sample was checked using an Agilent 2100 bioanalyzer (Agilent Technology Inc., Santa Clara, CA, USA). One microgram of qualified total RNA was used to construct the sequencing libraries. The NEB Next Ultra RNA LP Kit (New England Biolabs, Ipswich, MA, USA) was used for the construction. The library was sequenced using an Illumina NovaSeq 6000 to generate paired-end reads of 150 bp. Around 3 Gb raw reads were obtained for each sample. Adaptors were removed using Trim Galore. Processed reads were mapped to the mouse genome (UCSC mm10) using HISAT2^53–55^. The mapping results were visualized using an integrative genome viewer^56^. Transcripts Per Kilobase Million (TPM) were calculated using Stringtie based on the reference for genes (gencode: vM27. Annotation.gtf). Processed datasets (Accession No. GSE225883) are available from Gene Expression Omnibus (GEO; https://www.ncbi.nlm.nih.gov/geo/).

### Gene chip

The annotated 20,763 genes were analyzed using the Clariom S Assay (Thermo Fisher Scientific). At first, single-stranded cDNA was synthesized from total RNA (250 ng) extracted on day 27 under culture condition (iii) according to GeneChip™ WT PLUS Reagent Kit (Thermo Fischer Scientific). Then, fragmented cDNA was labeled with biotin. For hybridization, labeled cDNA was incubated with probes for 16 h at 45 °C. Signals were scanned by GeneChip™ Scanner 3000 7G (Thermo Fischer Scientific). Expression data from CEL files were normalized by the Robust Multi-Array Average method with R packages (“affy,” "pd.clariom.s.mouse," and "oligo")^57–59^. MA plots were generated based on fold change. Venn diagrams were drawn using the R package, “VennDiagram.” The distances of the genes were clustered into eight groups and visualized on a heatmap. Enrichplot packages were used to visualize the enriched GO terms and genes given by clusterProfiler and DOSE^60,61^. We disclosed the raw data (Accession No. GSE225882) from GEO.

### Immunofluorescence staining

Cultured cells were fixed with 4% paraformaldehyde (FUJIFILM Wako Pure Chemical Corporation, Osaka, Japan) and then incubated with PBS/Triton-X 100 (Nakalai Tesque) for permeabilization. For blocking, 5% bovine serum albumin (Sigma-Aldrich Inc.) was used. The cells were incubated with α-Nfatc1 antibody (Santa Cruz, 7A6, 1:50 dilution), α-Oscar antibody (Novus Biologica, Centennial, CO, USA, 5B8, 1:50), and α-Blimp1 antibody (Santa Cruz, 6D3, 1:50 dilution). Next, the cells were incubated with α-mouse IgG conjugated with Alexa Fluor 488 antibody (Thermo Fischer Scientific, A21200, 1:500 dilution) or α-rat IgG conjugated with Alexa Fluor 488 (Thermo Fischer Scientific, A21470, 1:500 dilution). Then, the cells were stained with Hoechst 33342 solution (FUJIFILM Wako Pure Chemical Corporation) and observed under a FluoView FV1000D inverted confocal microscope (Olympus).

### Supplementary Information

Supplementary Table S1.

Supplementary Table S2.

Supplementary Figures.

## Data Availability

The RNA-seq and Gene Chip data are available on Gene Expression Omnibus (GEO; https://www.ncbi.nlm.nih.gov/geo/).

## References

[1] Abboud SL, Woodruff K, Liu C, Shen V, Ghosh-Choudhury N (2002). Rescue of the osteopetrotic defect in op/op mice by osteoblast-specific targeting of soluble colony-stimulating factor-1. Endocrinology.

[2] Raisz LG (2005). Pathogenesis of osteoporosis: concepts, conflicts, and prospects. J. Clin. Invest..

[3] Hasegawa T (2019). Identification of a novel arthritis-associated osteoclast precursor macrophage regulated by FoxM1. Nat. Immunol..

[4] Győri DS, Mócsai A (2020). Osteoclast signal transduction during bone metastasis formation. Front. Cell Dev. Biol..

[5] Boyle WJ, Simonet WS, Lacey DL (2003). Osteoclast differentiation and activation. Nature.

[6] Mossadegh-Keller N (2013). M-CSF instructs myeloid lineage fate in single haematopoietic stem cells. Nature.

[7] Yasuda H (1998). Osteoclast differentiation factor is a ligand for osteoprotegerin/osteoclastogenesis-inhibitory factor and is identical to TRANCE/RANKL. Proc. Natl. Acad. Sci. USA.

[8] Fuller K, Wong B, Fox S, Choi Y, Chambers TJ (1998). TRANCE is necessary and sufficient for osteoblast-mediated activation of bone resorption in osteoclasts. J. Exp. Med..

[9] Lacey DL (1998). Osteoprotegerin ligand is a cytokine that regulates osteoclast differentiation and activation. Cell.

[10] Takayanagi H (2002). Induction and activation of the transcription factor NFATc1 (NFAT2) integrate RANKL signaling in terminal differentiation of osteoclasts. Dev. Cell.

[11] Asagiri M (2005). Autoamplification of NFATc1 expression determines its essential role in bone homeostasis. J. Exp. Med..

[12] Matsumoto M (2004). Essential role of p38 mitogen-activated protein kinase in cathepsin K gene expression during osteoclastogenesis through association of NFATc1 and PU.1. J. Biol. Chem..

[13] Matsuo K (2004). Nuclear factor of activated T-cells (NFAT) rescues osteoclastogenesis in precursors lacking c-Fos. J. Biol. Chem..

[14] Yagi M (2005). DC-STAMP is essential for cell–cell fusion in osteoclasts and foreign body giant cells. J. Exp. Med..

[15] Miyamoto H (2012). Osteoclast stimulatory transmembrane protein and dendritic cell-specific transmembrane protein cooperatively modulate cell–cell fusion to form osteoclasts and foreign body giant cells. J. Bone Miner. Res..

[16] de la Pompa JL (1998). Role of the NF-ATc transcription factor in morphogenesis of cardiac valves and septum. Nature.

[17] Ranger AM (1998). The transcription factor NF-ATc is essential for cardiac valve formation. Nature.

[18] Winslow MM (2006). Calcineurin/NFAT signaling in osteoblasts regulates bone mass. Dev. Cell.

[19] Aliprantis AO (2008). NFATc1 in mice represses osteoprotegerin during osteoclastogenesis and dissociates systemic osteopenia from inflammation in cherubism. J. Clin. Invest..

[20] Chuvpilo S (2002). Autoregulation of NFATc1/A expression facilitates effector T cells to escape from rapid apoptosis. Immunity.

[21] Serfling E, Chuvpilo S, Liu J, Höfer T, Palmetshofer A (2006). NFATc1 autoregulation: A crucial step for cell-fate determination. Trends Immunol..

[22] Wilkinson AC (2019). Long-term ex vivo haematopoietic-stem-cell expansion allows nonconditioned transplantation. Nature.

[23] Jin SH (2018). Actin-binding LIM protein 1 regulates receptor activator of NF-κB ligand-mediated osteoclast differentiation and motility. BMB Rep..

[24] Kim N, Takami M, Rho J, Josien R, Choi Y (2002). A novel member of the leukocyte receptor complex regulates osteoclast differentiation. J. Exp. Med..

[25] Narahara S (2019). KBTBD11, a novel BTB-Kelch protein, is a negative regulator of osteoclastogenesis through controlling Cullin3-mediated ubiquitination of NFATc1. Sci. Rep..

[26] Nayak A (2009). Sumoylation of the transcription factor NFATc1 leads to its subnuclear relocalization and interleukin-2 repression by histone deacetylase. J. Biol. Chem..

[27] Kim ET, Kwon KM, Lee MK, Park J, Ahn JH (2019). Sumoylation of a small isoform of NFATc1 is promoted by PIAS proteins and inhibits transactivation activity. Biochem. Biophys. Res. Commun..

[28] McGrath KE (2015). Distinct sources of hematopoietic progenitors emerge before HSCs and provide functional blood cells in the mammalian embryo. Cell Rep..

[29] Yokomizo T (2019). Hlf marks the developmental pathway for hematopoietic stem cells but not for erythro-myeloid progenitors. J. Exp. Med..

[30] Soares-da-Silva F (2021). Yolk sac, but not hematopoietic stem cell-derived progenitors, sustain erythropoiesis throughout murine embryonic life. J. Exp. Med..

[31] Hoeffel G (2015). C-Myb(+) erythro-myeloid progenitor-derived fetal monocytes give rise to adult tissue-resident macrophages. Immunity.

[32] Fleischer J (1996). Differential expression and function of CD80 (B7–1) and CD86 (B7–2) on human peripheral blood monocytes. Immunology.

[33] Dai XM (2002). Targeted disruption of the mouse colony-stimulating factor 1 receptor gene results in osteopetrosis, mononuclear phagocyte deficiency, increased primitive progenitor cell frequencies, and reproductive defects. Blood.

[34] Gordon S, Todd J, Cohn ZA (1974). In vitro synthesis and secretion of lysozyme by mononuclear phagocytes. J. Exp. Med..

[35] Arai F (1999). Commitment and differentiation of osteoclast precursor cells by the sequential expression of c-Fms and receptor activator of nuclear factor kappaB (RANK) receptors. J. Exp. Med..

[36] Barahona de Brito C, Klein-Hessling S, Serfling E, Patra AK (2022). Hematopoietic stem and progenitor cell maintenance and multiple lineage differentiation is an integral function of NFATc1. Cells.

[37] Lee BS (2018). Myosins in osteoclast formation and function. Biomolecules.

[38] Georgess D, Machuca-Gayet I, Blangy A, Jurdic P (2014). Podosome organization drives osteoclast-mediated bone resorption. Cell. Adh. Migr..

[39] Hayman AR, Bune AJ, Bradley JR, Rashbass J, Cox TM (2000). Osteoclastic tartrate-resistant acid phosphatase (Acp 5): Its localization to dendritic cells and diverse murine tissues. J. Histochem. Cytochem..

[40] Zenger S (2007). Proteolytic processing and polarized secretion of tartrate-resistant acid phosphatase is altered in a subpopulation of metaphyseal osteoclasts in cathepsin K-deficient mice. Bone.

[41] Kim Y (2005). Contribution of nuclear factor of activated T cells c1 to the transcriptional control of immunoreceptor osteoclast-associated receptor but not triggering receptor expressed by myeloid cells-2 during osteoclastogenesis. J. Biol. Chem..

[42] Kurihara N, Iwama A, Tatsumi J, Ikeda K, Suda T (1996). Macrophage-stimulating protein activates STK receptor tyrosine kinase on osteoclasts and facilitates bone resorption by osteoclast-like cells. Blood.

[43] Andrade K (2017). RON kinase: A target for treatment of cancer-induced bone destruction and osteoporosis. Sci. Transl. Med..

[44] Nishikawa K (2010). Blimp1-mediated repression of negative regulators is required for osteoclast differentiation. Proc. Natl. Acad. Sci. USA.

[45] Minami T (2004). Vascular endothelial growth factor- and thrombin-induced termination factor, Down syndrome critical region-1, attenuates endothelial cell proliferation and angiogenesis. J. Biol. Chem..

[46] Kim JH (2016). RCANs regulate the convergent roles of NFATc1 in bone homeostasis. Sci. Rep..

[47] Cho KO, Jeong KH, Cha JH, Kim SY (2020). Spatiotemporal expression of RCAN1 and its isoform RCAN1-4 in the mouse hippocampus after pilocarpine-induced status epilepticus. Korean J. Physiol. Pharmacol..

[48] Takayanagi H (2020). RANKL maintains bone homeostasis through c-Fos-dependent induction of interferon-beta. Nature..

[49] Stemmer M, Thumberger T, Del Sol Keyer M, Wittbrodt J, Mateo JL (2015). CCTop: An intuitive, flexible and reliable CRISPR/Cas9 target prediction tool. PLoS One.

[50] Yoshimi K (2016). ssODN-mediated knock-in with CRISPR-Cas for large genomic regions in zygotes. Nat. Commun..

[51] Sato K (2006). Regulation of osteoclast differentiation and function by the CaMK–CREB pathway. Nat. Med..

[52] Schneider CA, Rasband WS, Elicerri KW (2012). NIH Image to ImageJ: 25 years of image analysis. Nat. Methods.

[53] Li H (2009). The sequence alignment/map format and SAMtools. Bioinformatics.

[54] Kim D, Langmead B, Salzberg SL (2015). HISAT: A fast spliced aligner with low memory requirements. Nat. Methods.

[55] Pertea M, Kim D, Pertea GM, Leek JT, Salzberg SL (2016). Transcript-level expression analysis of RNA-seq experiments with HISAT, StringTie and Ballgown. Nat. Protoc..

[56] Robinson JT (2011). Integrative genomics viewer. Nat. Biotechnol..

[57] Irizarry RA (2003). Exploration, normalization, and summaries of high density oligonucleotide array probe level data. Biostatistics.

[58] Gautier L, Cope L, Bolstad BM, Irizarry RA (2004). affy—Analysis of Affymetrix GeneChip data at the probe level. Bioinformatics.

[59] Carvalho BS, Irizarry RA (2020). A framework for oligonucleotide microarray preprocessing. Bioinformatics.

[60] Yu G, Wang LG, Han Y, He QY (2012). clusterProfiler: An R package for comparing biological themes among gene clusters. OMICS.

[61] Yu G, Wang LG, Yan GR, He QY (2015). DOSE: An R/Bioconductor package for disease ontology semantic and enrichment analysis. Bioinformatics.

