# Supplementary material for: Essentiality of Nfatc1 short isoform in osteoclast differentiation and its self-regulation
Source: Sci Rep. 2023 Nov 1;13:18797. doi: 10.1038/s41598-023-45909-3 (PMC10620225; doi:10.1038/s41598-023-45909-3)
Supplement: Supplementary file 3 — Supplementary Figures. [file 41598_2023_45909_MOESM3_ESM.pdf]

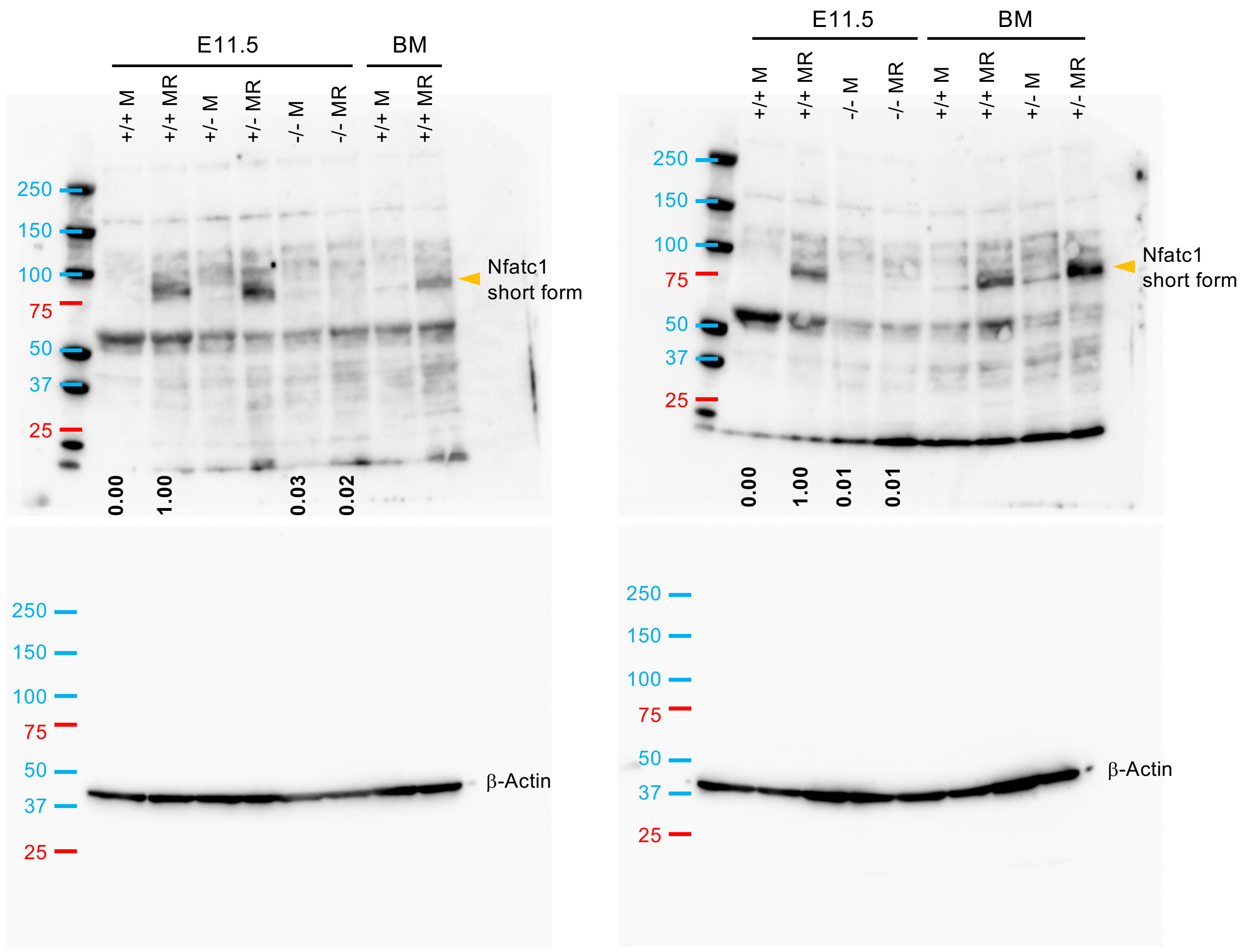

Supplementary Figure S1. Uncropped original blot images of blots shown in Figure 1b and Figure 3h. The full length blot for Nfatc1 (upper). Arrowheads indicate the Nfatc1 short form band. The same blot was reprobed with α-β-actin (lower). Relative band intensity of Nfatc1 short form in WT and KO culture is also shown.

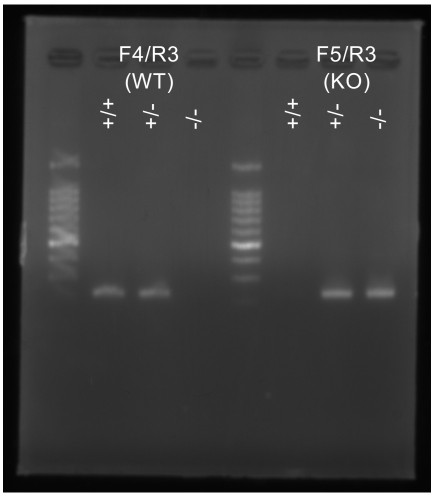

Supplementary Figure S2. An uncropped image of the agarose gel presented in Fig. 2d.

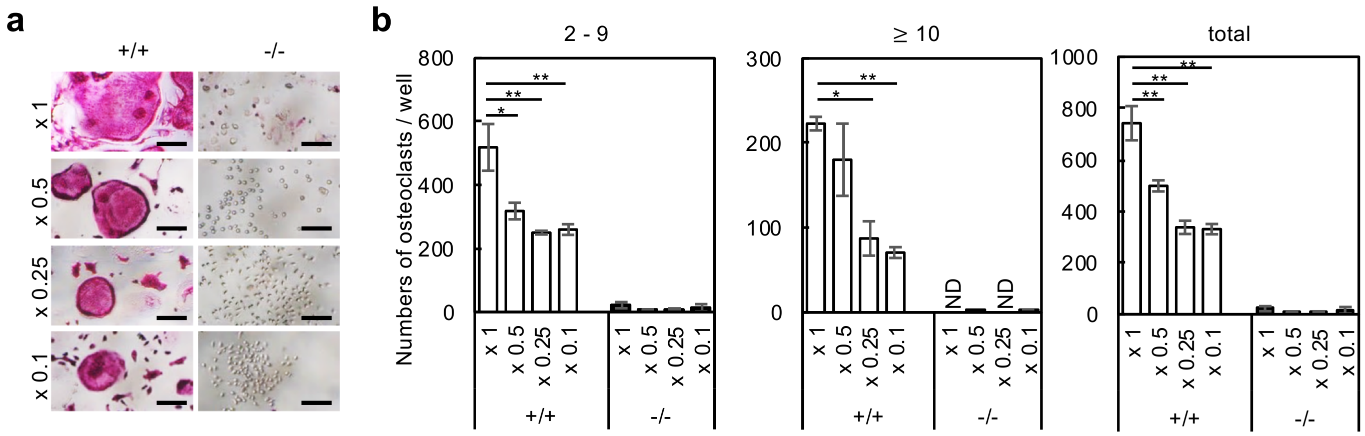

Supplementary Figure S3. Cell-density dependent osteoclast differentiation.

(a) TRAP staining of osteoclasts in culture from hematopoietic progenitors. Hematopoietic progenitors were seeded in a series of densities and cultured in condition (ii) (Fig. 3A) until day 31. Scale bars, 100 μm. **(b)** Number of osteoclasts differentiated from hematopoietic progenitors. All data are expressed as mean ± SE (n=3 technical replicates). Statistical significance was analyzed using the Tukey-Kramer test (vs. x1 (i), ** $p < 0.01$, * $p < 0.05$). At least four experiments were performed using hematopoietic progenitor cells from different embryos.

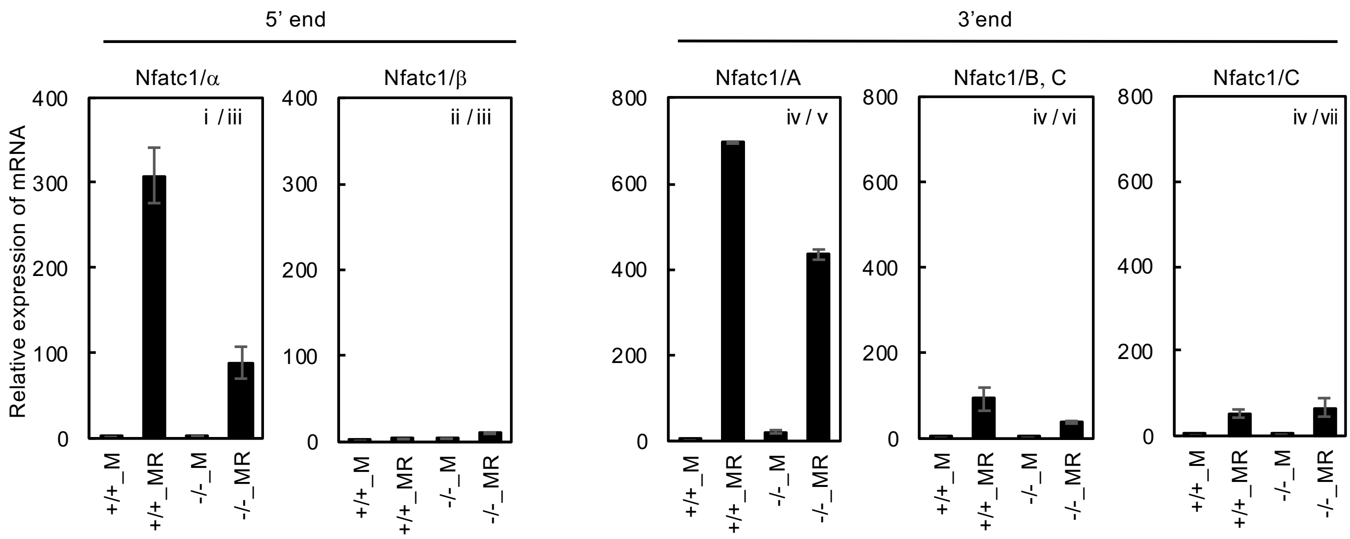

Supplementary Figure S4. Relative expression of Nfatc1 mRNA isoforms. Total RNA from cultured hematopoietic progenitors in condition (iii) on day 27 was analyzed by SYBR Green qRT-PCR. Primers specific to 5′ and 3′ regions were used for amplification.

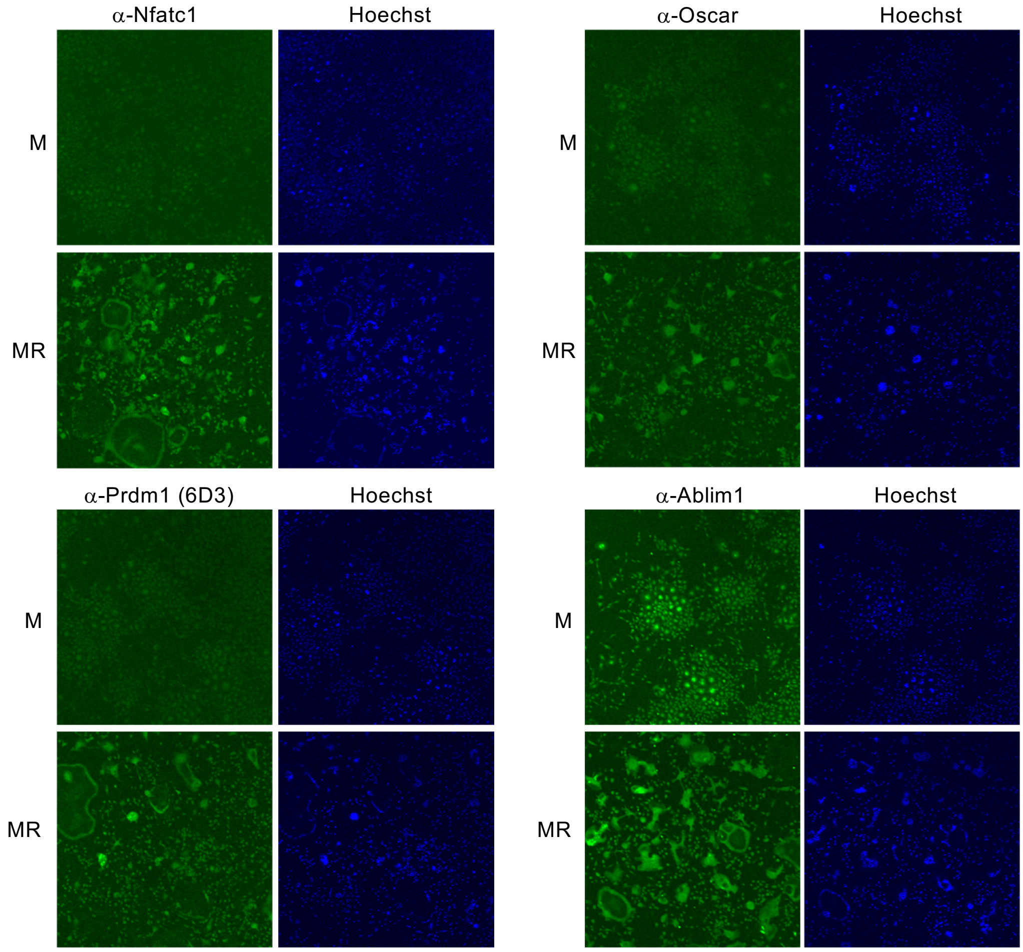

Supplementary Figure S5. Immunofluorescence staining of cultured bone marrow cells. Bone marrow cells from WT mice were cultured with M-CSF and RANKL for 3 days in 48-well plates. Fixed cells were stained with α-Nfatc1 antibody, α-Oscar antibody, α-Prdm1 antibody, α-Ablim1 antibody, and Hoechst.
